# Robust and Sustained Effect of Ketamine Infusions Coadministered with Conventional Antidepressants in a Patient with Refractory Major Depression

**DOI:** 10.1155/2015/815673

**Published:** 2015-03-04

**Authors:** José Manuel Montes, Elena Luján, Fernado Pascual, Jose María Beleña, Jose Luis Perez-Santar, Luis Javier Irastorza, Jerónimo Saiz-Ruiz

**Affiliations:** ^1^Psychiatry Service, Hospital Universitario Ramón y Cajal, Universidad de Alcalá, CIBERSAM, IRYCIS, Carretera Colmenar km. 9.1, 28034 Madrid, Spain; ^2^Psychiatry Service, Hospital Universitario del Sureste, Universidad Rey Juan Carlos, Ronda del Sur 10, 28500 Madrid, Spain; ^3^Anaesthesiology Service, Hospital Universitario del Sureste, Universidad Rey Juan Carlos, Ronda del Sur 10, 28500 Madrid, Spain

## Abstract

Antidepressant treatments show low capacity to achieve full clinical remissions. Electroconvulsive therapy is an alternative treatment which has been shown to be more effective but it is not well tolerated and there are concerns regarding its safety. We present the case of a patient with resistant depression and modest and transient response to ECT and who showed a robust and maintained response after six i.v. ketamine (0.5 mg/kg) infusions without withdrawing her antidepressant regimen. Ketamine was very well tolerated. This case illustrates the potential role of ketamine as a booster to standard antidepressants.

## 1. Introduction

Major depressive disorder (MDD) affects many people all over the world and is associated with severe personal and public health costs [1]. Current antidepressant treatments show low capacity to achieve full clinical remissions even after optimization of initial treatment [2, 3]. Furthermore, current antidepressants require a lag period of several weeks before an improvement is felt. Electroconvulsive therapy (ECT) is an alternative treatment to conventional drugs. ECT has been shown to be more effective than any other therapy [4] even in severe depression with psychotic symptoms. However, ECT is not well tolerated; there are concerns regarding its safety [4] and its use is limited to patients with contraindications to pharmacological treatments or who are treatment-resistant. Furthermore, ECT requires three weeks on average to cause a significant improvement in symptoms [5]. Ketamine is an anaesthetic agent which has shown a rapid and robust antidepressant effect after a single low-dose infusion, but results in ECT-resistant MDD are scarce and mixed [6–8]. Limitations for the use of ketamine in routine clinical practice include abuse potentiality and risk of illicit use. Additional concerns are related to its side effect profile (perceptual disturbances, dissociative states, or elevation in blood pressure) and difficulties to maintain sustained remission when used in monotherapy [9]. Herein, we present the case of a patient with MDD and a modest and transient response to ECT after standard agents failure and who showed a robust response with ketamine that could be maintained using psychotropic medications.

## 2. Case Presentation

The patient is a 56-year-old woman with a previous history of a major depressive episode 10 years ago. The first episode was precipitated by the diagnosis of renal cancer which required simple nephrectomy. After a failing trial with escitalopram, this first episode was treated with venlafaxine up to 300 mg/day during 16 weeks to obtain a sustained remission. All psychotropics were discontinued one year later and the patient maintained complete stability the next nine years. On January 2014 she was admitted to the psychiatric ward due to a relapse in severe depressive symptoms with continuous suicidal thoughts. The current episode might be triggered by a back pain that reminded the onset of the cancer. Imaging methods ruled out a cancer relapse and additional blood tests, including thyroid status, showed no significant abnormalities. The patient had a negative record for any substance use, but additional medical history included properly corrected hypothyroidism, intrinsic asthma, and hypercholesterolemia. Nevertheless, the initial anxiety symptoms generated by the fear of a cancer relapse gradually became a major depressive episode with extreme fear for everything, including any new physical or mental disease that kept her from going out of the house, restlessness, fragmented sleep, and lack of energy that caused total impairment of daily activities. Venlafaxine titrated to 300 mg/day had been initiated two weeks before admission with no relief. Quetiapine 400 mg/day was added and the patient showed an improvement in anxiety and insomnia but core depressive symptoms remained unmodified. After two more weeks without any improvement venlafaxine was switched to imipramine up to 300 mg/day and lithium at doses to obtain therapeutic levels. Three weeks later no improvement was observed and bilateral ECT was initiated three times per week until obtaining a slight improvement in mood that allowed discharge. The patient was complaining of moderate retrograde amnesia and subjective perception of mild cognitive deficit. During ECT sessions the medication regimen was switched to desvenlafaxine 100 mg/day, bupropion 300 mg/day, quetiapine 300 mg/day, and lamotrigine (100 mg/day). After a few days of staying at home the feelings of inability to cope with daily tasks were growing and provoked a new relapse in depression with a similar severity to the previous admission. Once in the psychiatric ward, lithium was added to her medication regimen while the institutional review board of the hospital approved the compassionate use of ketamine. Even with optimal serum lithium levels (0.7 mmol/L) the symptomatology remained unmodified and ketamine infusions were started two weeks later. The patient received six i.v. infusions of ketamine (0.5 mg/kg) during 40 min on a Monday-Thursday schedule over an 18-day period. This treatment was administered at the Post Anaesthesia Care Unit (PACU) under the supervision of a staff anaesthesiologist. Ketamine was very well tolerated and no changes in vital signs, including heart rate or blood pressure, were observed. Furthermore, no psychotomimetic or dissociative symptoms were experienced during all the infusions and cognitive complaints related to ECT did not get worse. All the psychotropic regimen was maintained with similar doses and there was no adverse effect associated with them. A robust improvement in mood was gradually observed after the third infusion allowing her to be discharged and gradually resuming her daily life activities. Six months later she is still in clinical remission and on the same medication regimen.

## 3. Discussion

This case illustrates the role of ketamine in achieving a relief of depressive symptoms in a patient with treatment-resistant depression, including a previous ECT trial not completely effective. The improvement obtained with ketamine was clearly higher than with ECT and associated with a greater tolerability. This is very important considering the dysfunctionality caused by the cognitive disturbances related to ECT.

Ketamine has shown robust and rapid antidepressant effect in randomized clinical trials, but one limitation for its use in routine clinical practice is the durability of the antidepressant response. A previous study has already shown the safety and efficacy of ketamine infusions without the acquisition of a medication-free state [10] for preventing the return of depressive symptoms as shown in this case. An alternative option to this approach is to find other routes of administration for ketamine. Ongoing clinical trials are assessing the oral and nasal administration of ketamine that could be helpful to maintain a sustained remission of depression.

In this clinical case the efficacy of ketamine could be attributed to the cumulative effect of the previous treatments. The two-week period from the second addition of lithium to the start of ketamine infusions could be considered very short. However, the poor response to the previous antidepressant treatments, including a previous trial with lithium, and the robust response observed after the third infusion support the role of ketamine in the improvement.

The neurobiological mechanisms underlying the antidepressant actions of ketamine remain unknown. The blockade of NMDA-type glutamate receptors is hypothesized to initiate a cascade in neurotrophic signalling and induce changes in synaptic plasticity. Thus, the robust antidepressant response observed in this case may be the result of a synergistic action of the modulation exerted by standard antidepressants on monoamine neurotransmission in conjunction with the effect of ketamine in the glutamate transmission. This opens the search of new compounds involving the glutamatergic system for the treatment of depression.

In the meantime, infusions of ketamine at low doses could be considered an alternative treatment to standard compounds in resistant MDD, including ECT-resistant, or when there is a need for a rapid relief of symptoms. Ketamine could be used as a booster to standard antidepressant regimens and safely administered without withdrawing medications. This procedure could help to avoid the return of depression observed with medication-free ketamine infusions.
